# Risk factors for HIV infection among married couples in Rakai, Uganda: a cross-sectional study

**DOI:** 10.1186/s12879-020-4924-0

**Published:** 2020-03-06

**Authors:** Anne M. Nabukenya, Aminah Nambuusi, Joseph K. B. Matovu

**Affiliations:** 1 MakSPH-CDC Fellowship Program, Makerere University School of Public Health, Kampala, Uganda; 2 Department of Social and Behavioral Sciences, Rakai Health Sciences Program, Kalisizo, Uganda; 3 Department of Disease Control and Environmental Health, Makerere University School of Public Health, P.O. Box 7072, Kampala, Uganda; 4 Department of Community and Public Health, Busitema University Faculty of Health Sciences, Busitema University, Mbale, Uganda

**Keywords:** Risk, HIV, Married couples, Rakai

## Abstract

**Background:**

Although married couples can be at an elevated risk of HIV infection, few studies have explored the risk factors for HIV infection at the couple-level. We explored the risk factors for HIV infection among married couples in settings with differing HIV prevalence levels in Rakai, Uganda.

**Methods:**

This was a cross-sectional study conducted among 664 heterosexual couples living in three HIV prevalence strata (low: 9–11.2%; medium: 11.4–20% or high HIV prevalence: 21–43%) in Rakai District, south-western Uganda, between November 2013 and February 2014. Data were collected on socio-demographic and behavioural characteristics from all consenting adults and aggregated to allow for couple-level analyses. We conducted bivariate and multivariable Logistic regression to assess the factors that were independently associated with HIV infection among married couples. Data were analysed using STATA statistical software (version 14.1).

**Results:**

Of the 664 couples, 6.4% (*n* = 42) were in HIV-discordant relationships; 5.8% (*n* = 39) were in concordant HIV-positive relationships while 87.8% (*n* = 583) were in concordant HIV-negative relationships. At the bivariate analysis, we found that residing in a high HIV prevalence stratum, reporting extra-marital relations, age difference between partners and number of previous marriages were significantly associated with being part of an HIV infected couple. After adjusting for potential cofounders, living in a high HIV prevalence stratum (Adjusted OR [AOR] =2.31, 95% CI: 1.52, 3.52), being in a third or higher order relationship (AOR = 3.80, 95% CI: 2.30, 6.28), and engagement in extra-marital relations (AOR = 1.75; 95% CI: 1.19, 2.59) were associated with couple HIV infection. Individuals that had stayed together for six or more years had 28% odds of being part of an HIV infected couple (AOR = 0.28; 95%CI: 0.18, 0.43).

**Conclusion:**

Living in a high HIV prevalence stratum, engagement in extra-marital relations and having a higher number of previous marriages were significant risk factors for HIV infection among married couples. Long marital duration was associated with reduced risk of HIV infection. Interventions that increase marital stability and those that promote pre-marital couples’ HIV testing before marital formation can reduce HIV transmission risk among married couples in this setting.

## Background

In 2017, 1.3 million people became newly infected with HIV worldwide [1]. Of these incident cases, approximately 75% occurred in sub-Saharan Africa. Uganda experienced 50,000 new HIV infections in the same year [1] and it was reported that HIV among adults aged 15 to 64 was 6.2%: 7.6% among females and 4.7% among males [2]. Several factors have been identified to explain the high HIV prevalence in sub-Saharan Africa including a history of transactional or paid sex; concurrent sexual partnerships, co-infection with viral and bacterial sexually transmitted infections (notably herpes simplex virus type 2), inconsistent or no condom use, lack of male circumcision among men, and marital status (widowed/divorced vs. never married) [3–6]. Studies have shown that in most SSA societies, over 80% of new HIV infections in women are estimated to occur in marriage or long-term relationships through heterosexual transmission [7, 8]. However, although heterosexual transmission has since been documented as a primary mode of HIV infection globally, previous studies have largely been conducted among individuals than couples presenting a missed opportunity for assessing risk factors for HIV infection among married and cohabiting individuals.

There are several reasons why being married may increase the risk of HIV infection, particularly in sub-Saharan communities where gender inequalities and high levels of masculinity norms inhibit access to HIV prevention and treatment services [9, 10]. For instance, men are more likely to report higher numbers of lifetime sexual partners and higher frequency of concurrency in sexual partnerships than women [11] and, although males report high consistent condom use than females, consistent use in regular relationships falls short of the level needed to protect them and their marital partners from the risk of HIV infection [11]. Gender inequalities, particularly in societies where it’s permissible for men to have extra marital relationships [12] render women to be particularly vulnerable to transmission from their husbands [13–15]. Besides, women living in rural areas are marginalized and disempowered and face geographical barriers in terms of accessing HIV knowledge and other HIV services [16]. Younger women married to older men do not have equal say in discussing safe sex practices [17, 18]. In addition, the risk of HIV acquisition is also known to be high among cohabiting or married couples especially when one of the partners is HIV positive [19] or where intimate partner violence exists [20, 21]. Collectively, these findings suggest that being married can increase the risk of heterosexual transmission of HIV particularly in sub-Saharan African settings where gender inequalities and high levels of background HIV prevalence combine to make married individuals susceptible to HIV infection. However, few studies have explored the risk factors for HIV infection within marital unions. The objective of this study was to expand current literature on HIV infection among married individuals by assessing the risk factors for HIV infection among married couples in a setting where HIV infection is higher than the national average.

## Methods

### Study design and population

The paper uses data from a large cross-sectional study conducted among married and cohabiting individuals in Rakai district, south western Uganda between November 2013 and February 2014 [22]. The dataset contains 1834 unique individuals with known HIV status. These data were merged using partner identification information to form 664 complete couples. Individuals were sampled from three study regions of differing HIV prevalence (range: 9–43%) within the Rakai Community Cohort Study (RCCS) enumeration area. The RCCS has been previously described [23, 24].

In brief, the RCCS is a population-based study with approximately annual surveys of 14,000 consenting persons aged 15–49 years, resident in 50 communities, and has been ongoing since 1994 and has been described elsewhere. Census is done prior to each survey round to identify eligible participants who are then contacted in their homes or invited to attend at central locations (“hubs”) for interview and provision of blood for HIV diagnosis. Interviews are then done to ascertain information on socio demographic characteristics, sexual behaviors and health every 12 to 18 months using structured questionnaires administered in private by same sex interviewers. The large study from which these data have been drawn was conducted within the context of the RCCS. Based on available data, the pooled estimate of HIV prevalence across the three study regions was 23.2%.

### Sampling procedures

Initially, all the eleven study regions that form the RCCS enumeration areas were grouped into three categories based on HIV prevalence data from the RCCS. The decision on the lower and upper boundaries for each stratum was made by the study investigators at the time of study initiation. The lowest HIV prevalence was 9% while the highest was 43%. The study regions were thus grouped into low HIV prevalence (9–11.2%), medium prevalence (11.4–20%) or high HIV prevalence regions (21–43%). The grouping of study regions into the three strata was done in such a way as to ensure that each stratum had between 3 and 4 study regions. Within each stratum, one region was selected to participate in the study. Within each study region, four study communities were randomly selected using computer-generated random numbers for a total of 12 study communities. The study communities were already demarcated for their participation in the RCCS; so, there was no need for further demarcation. Residents in the selected communities who were aged 15–49 years and who were married or in a cohabiting relationship at the time of the study were eligible for inclusion in the study.

### Data collection procedures and methods

Data were collected using interviewer-administered questionnaires. Data were collected on socio-demographic (age, sex, education, religion) and behavioural (condom use at last sex, non-marital sexual relationships, number of sexual partners in the past 12 months, and alcohol use before sex) characteristics. Prior to the interviews, individuals were invited to a “central hub” – a location within the community that individuals considered to be within easy reach by all participants. Individuals who did not turn up at the hub were followed up at home, and if available, they were interviewed. Data collection within each stratum took, on average, up to 4 weeks. All individuals gave informed written informed consent prior to participation in the study. Interviews, on average, lasted between 45 and 60 min. To ensure that individuals would be easily linked to their marital partners, partner identifying information (e.g. name) were obtained from each interviewed respondent. Individuals were then linked to their marital partners using study identifiers.

### Measurement of variables

We used the term ‘married couples’ to refer to individuals who, in response to two pre-set questions (“*are you currently married*?”; if yes, “*what type of marital union are you currently engaged in?*”), responded that they were either ‘officially’ married in church or mosque; had a traditional introduction ceremony done; were married at the marriage registrar’s office; or were in a consensual union – defined as a union in which both members considered themselves as married and were also considered as ‘married’ by the community in which they lived. Our analysis focused on individuals who were in a heterosexual relationship; i.e. currently married individuals with an identifiable partner of the opposite sex. We used the term ‘marital order’ to refer to the number of times an individual has ever been married, counting from their current marital relationship. Individuals that reported that they had never been in any other marital relationship other than the current one were categorized as being in their ‘first’ union while those that had ever been in any previous relationship that ended were categorized as being in their ‘second’ or ‘third or higher-order’ union, depending on the number of times that they had been previously married. The outcome variable, the HIV infected couple, is hereby defined as a couple where one or both partners were HIV positive. An individual was classified as living as part of an HIV infected couple or HIV positive couple relationship if he or she was positive or the partner was positive status, obtained by linking individuals in a couple who were either in an HIV discordant relationship or HIV concordant relationship. A couple was defined as polygamous if a man indicated that he had more than one wife (married or cohabiting) or a woman indicated that her male partner had more than one wife.

### Statistical analysis

The dependant variable was binary whether an individual is living as part of an infected couple or not. This was summarised using frequencies and percentages. Similarly, all categorical independent variables were summarized using frequencies and percentages. Unadjusted Odds ratios and their 95% Confidence intervals were used to assess the association between HIV infection and different potential risk factors at the bivariate analysis level. Only factors that had a likelihood ratio test *p*-value < 0.02 were included in the multivariable logistic regression. Data were analysed using STATA statistical software (version 14.1).

## Results

### Sample characteristics

Data were obtained from a sample of 1314 respondents living as part of 664 heterosexual couples. In 46% (*n* = 305) of the couples, the man was at least 6 years older than the woman while in 7% (*n* = 46) of the couples, the woman was older than the man. Of the 1314 individuals, 18.4% were in polygamous relationships; 88% had been together for at least 5 years; while 34.4% reported the current relationship to be their second, third or higher-order marriage (Table 1). Sixty-six per cent (66%, *n* = 867) of the respondents were aged between 25 and 39 years while 25.2% (*n* = 331) had secondary school education or higher. Majority of the individuals (59.5%, *n* = 788) were Roman Catholics.

**Table 1:** Socio-demographic characteristics of 1314 married or cohabiting individuals

Characteristics	Total	Percentage
**Age-group**		
15–24 years	221	16.8
25–29 years	325	24.7
30–34 years	289	22.0
35–39 years	258	19.6
40+ years	221	16.8
**Education level**		
None	76	5.8
Lower primary (P1-P4)	266	20.2
Upper primary (P5-P7)	641	48.8
Secondary & above	331	25.2
**Religion**		
Catholic	782	59.5
Protestant	178	13.5
Saved/Pentecostal	81	6.2
Muslim	242	18.4
Other	31	2.4
**Marital order**		
First	862	65.6
Second	321	24.4
Third or higher	131	10.0
**Marital duration**		
1–3 years	157	11.9
4–5 years	167	12.7
6+ years	990	75.3
**Age at first marriage**		
11–17 years	323	24.6
18–20 years	500	38.1
21–24 years	255	19.4
25–29 years	188	14.3
30+ years	48	3.7
**Ever use of condoms**		
Yes	842	64.1
No	472	35.9
**In polygamy**		
No	1064	81.0
Yes	242	18.4
Don’t know	8	0.6
**Age difference of the couple**^**a**^		
Same age	186	14.7
Woman older	87	6.9
Man 2–5 years	414	32.7
Man 6–10 years	390	30.8
Man 11+ years	188	14.9
**More than one sexual partner**		
No	1102	83.9
Yes	212	16.1

^a^Expressed out of 1265 individuals for whom complete data on age difference was available

### HIV prevalence and associated risk factors

Of the 664 couples, 6.4% (*n* = 42) were in HIV-discordant relationships; 5.8% (*n* = 39) were in concordant HIV-positive relationships (in which both partners were HIV-positive) while 87.8% (*n* = 583) were in concordant HIV-negative relationships. Of those in HIV-discordant relationships, 52.4% (*n* = 22) had the male partner HIV-infected while 47.6% (*n* = 20) had the female partner HIV-infected. Overall, 12.2% (*n* = 81) of couples had at least one HIV-infected partner (data not shown).

Table 2 shows bivariate analysis of risk factors for HIV infection among couples. Couples resident in a high HIV prevalence region had 4 times the odds of living as part of an HIV infected couple (OR = 4.29; 95% CI: 2.95, 6.25) than those living in a medium or low HIV prevalence region. Similarly, respondents who have had one previous couple relationship had about 4 times the odds of living in an HIV infected couple (OR = 4.28; 95%CI: 3.10, 5.90) while those who had at least two previous couple relationships had 6 times the odds of being in an HIV infected couple (OR = 6.26; 95%: 4.12, 9.48). In other words, the number of previous couple dissolutions was a strong predictor of HIV infection in the current couple. Related to this, the risk of HIV infection in the couple reduces with the number of years lived together. Individuals in a couple that has been together for at least 6 years had only 16% odds of being HIV positive as compared to those who have lived together for three or less years (OR = 0.16; 95%CI: 0.11, 0.23). Table 2 also shows that age difference between partners in a couple was a significant risk factor for HIV infection. In couples where a woman was older, the odds of HIV infection were twice as high as where partners were of similar age (OR = 2.13; 95%CI: 1.17, 3.88). Similarly, involvement in risk sex was strongly associated with HIV infection among couples. Individuals who had more than one sexual partner in the past 12 months had about twice the odds of living as part of an HIV infected couple (OR = 1.88; 95%CI: 1.31, 2.61) as those who were not engaged in such relationships.

**Table 2:** Bivariate analysis of risk factors for HIV infection among couples

Factor	Overall Sample	HIV positive sample			Unadjusted ORs (95% CI)		*p*-value
	N	*n*	%	95% CI	OR	95% CI	
**HIV prevalence strata**							
Medium	433	40	9.2	(6.5, 12.0)	1.00		
Low	371	22	5.9	(3.5, 8.3)	0.62	(0.36, 1.06)	0.082
High	510	155	30.4	(26.4, 34.4)	4.29	(2.95, 6.25)	0.000
**Age-group**							
15–24 years	221	42	19.0	(13.8, 24.2)	1.00		
25–29 years	325	59	18.2	(14.0, 22.4)	0.95	(0.61, 1.47)	0.802
30–34 years	289	54	18.7	(14.2, 23.2)	0.98	(0.63, 1.53)	0.927
35–39 years	258	30	11.6	(7.7, 15.6)	0.56	(0.34, 0.93)	0.026
40+ years	221	32	14.5	(9.8, 19.1)	0.72	(0.44, 1.19)	0.204
**Education level**							
None	76	15	19.7	(10.6, 28.9)	1.00		
Lower primary	266	48	18.0	(13.3, 22.8)	0.90	(0.50, 1.79)	0.776
Upper primary	641	105	16.4	(13.5, 19.3)	0.80	(0.44, 1.55)	0.495
Secondary & above	331	49	14.8	(10.9, 18.7)	0.71	(0.39, 1.47)	0.432
**Religion**							
Catholic	782	139	17.8	(15.1, 20.5)	1.00		
Protestant	178	33	18.5	(12.8, 24.3)	1.05	(0.69, 1.6)	0.810
Saved/Pentecostal	81	12	14.8	(7.0, 22.6)	0.80	(0.43, 1.53)	0.505
Muslim	242	25	10.3	(6.5, 14.2)	0.53	(0.34, 0.84)	0.006
Other	31	8	25.8	(10.1, 41.5)	1.61	(0.71, 3.67)	0.259
**Marital order**							
First	862	76	9.3	(6.9, 10.7)	1.00		
Second	321	93	30.5	(23.9, 34.0)	4.28	(3.10, 5.90)	0.000
Third or higher	131	48	39.1	(28.2, 45.1)	6.26	(4.12, 9.48)	0.000
**Marital duration**							
1–3 years	157	69	43.9	(36.2, 51.7)			
4–5 years	167	40	24.0	(17.5, 30.5)	0.40	(0.25, 0.65)	0.000
6+ years	990	108	10.9	(9.0, 12.9)	0.16	(0.11, 0.23)	0.000
**Age at first marriage**							
11–17 years	323	58	18	(13.8, 22.2)	1.00		
18–20 years	500	75	15	(11.9, 18.1)	0.81	(0.55, 1.17)	0.261
21–24 years	255	46	18	(13.3, 22.8)	1.01	(0.66, 1.54)	0.980
25–29 years	188	30	16	(10.7, 21.2)	0.87	(0.54, 1.41)	0.564
30+ years	48	8	16.7	(6.0, 27.3)	0.91	(0.41, 2.06)	0.827
**Ever use of condoms**							
Yes	842	166	19.7	(17.0, 22.4)	1.00		
No	472	51	10.8	(8.0, 13.6)	0.49	(0.35, 0.69)	0.000
**In polygamy**							
No	1064	173	16.3	(14.0, 18.5)	1.00		
Yes	242	42	17.4	(12.6, 22.1)	1.08	(0.75, 1.57)	0.678
Don’t know	8	2	25.0	(0.0, 50.1)	1.72	(0.34, 8.58)	0.510
**Age difference of the couple**							
Same age	186	31	16.7	(11.3, 22.0)	1.00		
Woman older	87	26	29.9	(20.2, 39.6)	2.13	(1.17, 3.88)	0.013
Man 2–5 years	414	57	13.8	(10.4, 17.1)	0.79	(0.5, 1.29)	0.354
Man 6–10 years	390	22	5.6	(3.3, 7.9)	0.51	(0.32, 0.83)	0.006
Man 11+ years	188	32	17.0	(11.6, 22.4)			
**More than one sexual partner**							
No	1102	162	14.70	(12.6, 16.8)	1.00		
Yes	212	55	25.94	(20.0, 32.0)	1.88	(1.31, 2.61)	0.000

Table 3 shows the results from the multivariable analysis. Region of residence, number of previous couple unions, marital duration and possession of more than one sexual partners were associated with HIV infection among couples. Couples in areas of high HIV prevalence were twice as likely to have HIV infection (AOR = 2.31; 95% CI: 1.52, 3.52) as those in low HIV prevalence areas. Individuals who had three or more previous couple unions had nearly four times the odds of being part of an HIV infected couple compared to those with only the current union as the only union they had ever had (AOR = 3.80; 95%CI: 2.30, 6.28). Further, individuals that have been together for more than 6 years had 28% odds of being part of an HIV infected couple as compared to those who have lived together for three or less years (AOR = 0.28; 95%CI: 0.18, 0.43). Lastly, individuals with more sexual partners were almost twice more likely to be in an infected couple relationship than those in single partner relationships ((AOR = 1.75; 95% CI: 1.19, 2.59).

**Table 3:** Multivariable logistic model for risk factors for HIV infection among couples in Rakai, Uganda

Factor	Overall sample	HIV positive sample			Adjusted ORs (95% CI)		*p*-value
	N	*n*	%	95% CI	ORs	95% CI	
**HIV prevalence strata**							
Medium	433	40	9.2	(6.5, 12.0)	1.00		
Low	371	22	5.9	(3.5, 8.3)	0.62	(0.33, 1.02)	0.055
High	510	155	30.4	(26.4, 34.4)	2.31	(1.52, 3.52)	0.000
**Marital order**							
First	862	76	9.3	(6.9, 10.7)	1.00		
Second	321	93	30.5	(23.9, 34.0)	3.34	(2.32, 4.70)	0.000
Third or higher	131	48	39.1	(28.2, 45.1)	3.80	(2.30, 6.28)	0.000
**Marital duration**							
1–3 years	157	69	43.9	(36.2, 51.7)			
4–5 years	167	40	24.0	(17.5, 30.5)	0.64	(0.38, 0.71)	0.093
6+ years	990	108	10.9	(9.0, 12.9)	0.28	(0.18, 0.43)	0.000
**More than one sexual partner**							
No	1102	162	14.70	(12.6, 16.8)	1.00		
Yes	212	55	25.94	(20.0, 32.0)	1.75	(1.19, 2.59)	0.006

## Discussion

In this study, we investigated the risk factors for HIV infection among married couples in Rakai, Uganda. The key factors associated with HIV infection among the couples included residing in a high HIV prevalence study region, the increasing number of the previous couple unions an individual has had, and one’s sexual involvement with more than one partner. After adjusting for potential and suspected confounders, we found that individuals residing in a high HIV prevalence region were twice as likely to be infected with HIV as those living in medium or low HIV prevalence settings. Similarly, individuals who had been in three or more previous couple relationships were almost four times more likely to be infected with HIV than those in the first marital union while individuals who reported having more than one sexual partner were almost two times more likely to be in an HIV-infected relationship than those who reported that they had only one sexual partner.

Similar to our findings, a study in Kenya reported that proportions of couples affected with HIV were highest in areas with highest HIV prevalence such as Nyanza [19]. The higher the prevalence of HIV in the community, the higher the chances that one marries an HIV positive partner or gets infected when involved in extra-marital sex. Collectively, these findings suggest that massive sensitization on HIV awareness in communities is still necessary to attract residents to test, seek treatment and prevention services. This should remind the government and its HIV implementing partners that the lower the HIV prevalence in the community or country, the less the chances of transmission. Hence, there is a need to encourage pre-marital couple testing and counseling in regions with high HIV prevalence.

The association between the number of previous couple unions and HIV infection among couples was also reported by Walque and Kline [25] who found that remarriages are associated with high risk of HIV acquisition. A previous Ugandan study reported higher HIV incidence among men and women who were in their second or higher order marital unions than those in first marital unions [26]. In some cases, individuals form new marital unions after loss of a partner or even after separation from the partner due to HIV infection but rarely do they test for HIV prior to marital formation. Also, as indicated in the results, the number of previous marital unions may be a risk factor for an individual to acquire HIV. According to Żaba et al. [26], the earlier an individual starts sexual activity, the bigger the exposure to sexually transmitted infections including HIV. This is because at the time they choose to settle with a single partner for life, they may have already acquired HIV from their previous relationships, posing a risk to their subsequent partners.

Sexual relationships that occur between two people of different age brackets is a risk factor as seen from the results and it is common in many parts of the world [17, 18]. Usually, these relationships are embraced for the benefit of material support and other gains to the women and for pleasure to the men [27]. However, even the reverse is true for young men; there is a big number of young men who engage in sex with older women for the benefit of financial gain to the young men and satisfaction to the older women [28]. Transactional sex with older men or older women is a predisposing factor for young women and men respectively because of the unsafe behaviour involved in it with little or no command for safety precautions [29]. An age-disparate sex and HIV risk study for young women in South Africa, reported that majority of sexually active adolescent girls have experienced a relationship with an older man at least 5 years older than them. As a result, the cycle of transmission among this category of women is always driven by older men [28].

### Strengths and limitations

A key strength for this study is that it was conducted among study regions with differing HIV prevalence levels giving us the opportunity to adjust for background HIV prevalence while assessing the risk factors for HIV infection among married couples. However, since this was a cross-sectional study, it is difficult to tell whether the risk factors precluded the observed HIV infection in married couples or whether the observed risk factors emerged as a result of HIV infection. For instance, we can’t tell if individuals who had four or more previous relationships were infected as they moved from one relationship to the other or whether it was because of HIV infection that they moved from one relationship to the other, possibly after marital dissolution. The other limitation pertains to the fact that we conducted interviewer-administered interviews which may have created challenges for respondents in responding to sensitive questions, e.g. questions on sexual behaviours. To address these challenges, we trained all interviewers in how to conduct interviews involving sensitive questions and, although we targeted couples, we ensured that the interviews were conducted separately for each partner to allow for individuals to respond to sensitive questions. Nevertheless, these limitations notwithstanding, our study provides information that is necessary to inform interventions targeting married couples, particularly the need to promote pre-marital HIV counselling and testing among individuals living in high HIV prevalence communities and those intending to remarry after the dissolution of a previous marriage. Such interventions are urgently needed to reduce the risk of HIV infection among married couples.

## Conclusion

In conclusion, our study of risk factors for HIV infection among married couples in Rakai district, southwestern Uganda, found that living in a high HIV prevalence stratum, engagement in extra-marital relations and having a higher number of previous marital relationships were significant risk factors for HIV infection among married couples in this part of the world. We also found that long marital duration was associated with reduced risk of HIV infection. These findings suggest a need for interventions that promote marital stability among currently married couples on the one hand, and, on the other hand, intensified efforts to promote pre-marital counseling before marital formation particularly among individuals who intend to remarry after the dissolution of their previous unions in order to reduce the risk of HIV transmission in their subsequent relationships.

## Data Availability

The datasets used and/or analyzed during the current study are available from the corresponding author on reasonable request.

## Abbreviations

AOR: Adjusted Odds Ratio
CI: Confidence Interval
HIV: Human Immunodeficiency Virus
OR: Odds Ratio
RCCS: Rakai Community Cohort Study
REC: Research and Ethics Committee
UVRI: Uganda Virus Research Institute

## References

[1] UNAIDS 'AIDSinfo'. http://www.avert.org. Accessed 4 Sept 2019.

[2] WHO/Uganda Ministry of Health. https://www.afro.who.int/sites/default/files/2017-08/UPHIA%20Uganda%20factsheet.pdf. Accessed 4 Sept 2019.

[3] Chen L, Jha P, Stirling B, Sgaier SK, Daid T, Kaul R, Nagelkerke N (2007). Sexual risk factors for HIV infection in early and advanced HIV epidemics in sub-Saharan Africa. Systematic overview of 68 epidemiological studies. PLoS One.

[4] Aj K (2013). Marital status and HIV/AIDS mortality: evidence from the US National Longitudinal Mortality study. Int J Infect Dis.

[5] Shisana O, Toefy Y, Simbayi LC, Malik S, Zuma K (2004). Marital status and HIV infection in South Africa. S Afr Med J.

[6] Shisana O, Rehle T, Simbayi LC, Zuma K, Jooste S, Zungu N (2014). South African National HIV prevalence, incidence and behaviour survey, 2012.

[7] Dunkle L, Stephenson R, Karita E (2008). New heterosexually transmitted HIV infections in married or cohabiting couples in urban Zambia and Rwanda: an analysis of survey and clinical data. Lancet.

[8] Malamba S, Mermin J, Bunnell R (2005). Couples at Risk: HIV-1 Concordance and Discordance Among Sexual Partners Receiving Voluntary Counseling and Testing in Uganda. J Acquir Immune Defic Syndr.

[9] Musheke M, Merten S, Bond V (2016). Why do marital partners of people living with HIV not test for HIV? A qualitative study in Lusaka, Zambia. BMC Public Health.

[10] Mtenga S, Pfeiffer C, Sonja M (2015). Prevalence and social drivers of HIV among married and cohabitating heterosexual adults in south-eastern Tanzania: analysis of adult health community cohort data. Glob Health Action.

[11] Genberg BL, Kulich M, Kawichai S (2008). HIV Risk Behaviors in Sub-Saharan Africa and Northern Thailand: Baseline Behavioral Data from Project Accept. J Acquir Immune Defic Syndr.

[12] Auerbach JD, Parkhurst JO, Caceres CF (2011). Addressing social drivers of HIV/AIDS for the long-term response: conceptual and methodological considerations. Global Public Health.

[13] Shisana O, Labadarios D, Simbayi L (2015). South African national HIV prevalence, Incidence and behaviour survey, 2012.

[14] Abdulazeez A, Alo E, Naphthali R (2008). Concurrent infection of HIV-! And HIV-2 serotypes in Adamawa State Nigeria. World J Med Sci.

[15] Joint United Nations Programme on HIV/AIDS (UNAIDS) and the World Health Organisation (WHO) (2009). AIDS epidemic update 2009.

[16] Bandali S (2014). Women Living with HIV in Rural Areas. Implementing a Response using the HIV and AIDS Risk Assessment and Reduction Model. Clin Med Insights Women’s Health.

[17] Gilbert L, Walker L (2002). Treading the path of least resistance: HIV/AIDS and social inequalities - A South African case study. Soc Sci Med.

[18] Bandali S (2013). HIV risk assessment and risk reduction strategies in the context of prevailing gender norms in rural areas of Cabo Delgado, Mozambique. JIAPAC.

[19] Kaiser R, Bunnell R, Hightower A, Kim AA, Cherutich P, Mwangi M (2011). Factors Associated with HIV Infection in Married or Cohabitating Couples in Kenya: Results from a nationally representative study. PLoS One.

[20] Mkandawire L, Wendland C, Stevens PE (2013). Marriage as a risk factor for HIV: learning from the experiences of HIV-infected women in Malawi. Glob Public Health.

[21] Langen TT (2005). Gender power imbalance on women's capacity to negotiate self-protection against HIV/AIDS in Botswana and South Africa. Afr Health Sci.

[22] Matovu JK, Jim T, Wanyenze R (2015). Correlates of previous couples’ HIV counseling and testing uptake among married individuals in three HIV prevalence strata in Rakai, Uganda. Glob Health Action.

[23] Wawer MJ, Gray RH, Sewankambo NK (1999). A randomized community trial of intensive sexually transmitted disease for control of AIDS prevention Rakai, Uganda. A randomized community trial. Lancet.

[24] Grabowski MK, Reynolds SJ, Kagaayi J (2018). The validity of self-reported antiretroviral use in persons living with HIV: a population-based study. AIDS.

[25] Walque W, Kline R (2012). The Association Between Remarriage and HIV Infection in 13 Sub-Saharan African Countries. Stud Fam Plann.

[26] Żaba B, Isingo R, Wringe A (2009). Influence of timing of sexual debut and first marriage on sexual behaviour in later life: findings from four survey rounds in the Kisesa cohort in northern Tanzania. Sex Transm Infect.

[27] Stoebenaua K, Heiseb L, Wamoyic J, Bobrovad N (2016). Revisiting the understanding of “transactional sex” in sub-Saharan Africa: A review and synthesis of the literature’. Soc Sci Med.

[28] Minki C, Nancy M (2004). The factors influencing transactional sex among young men and women in 12 Sub-Saharan African countries. J Soc Biol.

[29] ATHENA (2013). Integrating strategies to address gender-based violence and engage men and boys as partners to advance gender equality through National Strategic Plans on HIV and AIDS: West and Central Africa Regional Consultation.

